# Type 1 classical dendritic cells govern long-term cardiac allograft acceptance

**DOI:** 10.1172/JCI192811

**Published:** 2025-07-08

**Authors:** Macee C. Owen, Vinay R. Penna, Hao Dun, Wenjun Li, Benjamin J. Kopecky, Kenneth M. Murphy, Daniel Kreisel, Kory J. Lavine

**Affiliations:** 1 Department of Medicine, Washington University School of Medicine, St. Louis, Missouri, USA.; 2 Department of Medicine, University of Colorado Anschutz Medical Campus, Aurora, Colorado, USA.; 3 Department of Surgery,; 4 Department of Pathology & Immunology, and; 5 Department of Developmental Biology, Washington University School of Medicine, St. Louis, Missouri, USA.

**Keywords:** Cardiology, Inflammation, Organ transplantation

**To the Editor: **Cardiac transplantation is a lifesaving procedure for patients with complex congenital heart diseases and end-stage heart failure. Unfortunately, rejection remains common owing to limitations in current immunosuppressive strategies and alternative therapies are needed. Among proposed strategies, costimulation blockade (CSB) represents a promising approach, promoting tolerance rather than suppressing alloimmune responses. CSB with CTLA4-Ig and anti-CD40L antibodies is efficacious in experimental models and early clinical studies in islet and kidney transplantation (1, 2). How CSB modulates recipient immune responses remains incompletely understood.

Costimulation pathways signal bidirectionally, influencing both antigen-presenting cells (APCs) and T cells. While CSB’s effect on T cells is well studied, less is known about its effects on APCs. To investigate how CSB and a conventional immunosuppressant (cyclosporine [CSA]) influence APCs in cardiac allografts, we performed single-cell RNA sequencing (scRNA-Seq) on murine hearts 7 days after transplant. BALB/c donor hearts were transplanted into B6 *Zbtb46^gfp/+^* recipients treated with either CSB (anti-CD40L and CTLA4-Ig) or CSA. Histologically, CSA-treated grafts exhibited increased cellular infiltration compared with CSB-treated counterparts (Supplemental Figure 1A; supplemental material available online with this article; https://doi.org/10.1172/JCI192811DS1). Flow cytometry–isolated mononuclear phagocytes were used for 10X Genomics scRNA-seq, yielding 14,524 high-quality cells (Supplemental Figure 1, B and C), including monocyte, macrophage, and classical DC (cDC) subsets (Figure 1A and Supplemental Figure 1, D and E). CSB-treated grafts were enriched for recipient cDCs (GFP^+^), whereas CSA-treated grafts had increased monocytes and macrophages (Figure 1B). Reference mapping of naive hearts, syngeneic grafts, and a second model of allograft rejection (low-dose CTLA4-Ig) highlighted that cDC enrichment was CSB specific (Supplemental Figure 1F). Differential gene expression analysis revealed upregulation of genes involved in cDC activation, antigen presentation, and immunoregulation in CSB samples (Supplemental Figure 1, G and H). Flow cytometry and immunostaining confirmed increased frequencies of GFP^+^ cDCs in CSB-treated allografts, with a shift toward higher proportions of type 1 cDCs (cDC1s) (Figure 1, C–E, and Supplemental Figure 2A). Moreover, cDCs in CSB-treated allografts expressed PDL1 at a higher frequency than cDCs in CSA-treated allografts (Figure 1, D and E).

We next set out to define the requirement for recipient cDC1s and cDC2s in CSB-mediated long-term cardiac allograft acceptance. BALB/c hearts were transplanted into WT, Δ1+2+3 (cDC2-deficient), and *Irf8+32^–/–^* (cDC1-deficient) B6 CSB-treated recipients (Supplemental Figure 2B) (3, 4). While WT and cDC2-deficient recipients accepted cardiac allografts long-term, cDC1-deficient recipients rejected the transplanted hearts (Figure 1, F and G). *Irf8+32^–/–^* recipients exhibited intragraft infiltration of CD4^+^ and CD8^+^ T cells at day 14 after transplant and the time of rejection. WT and Δ1+2+3 recipients had significantly fewer T cells at both time points (Figure 1H and Supplemental Figure 2C). We also observed increased Foxp3^+^CD4^+^ T cells in allografts transplanted into WT versus *Irf8+32^–/–^* recipients, suggesting that cDC1s recruit regulatory T cells (Supplemental Figure 2D).

To examine if cDC1 deficiency impacts the composition and transcriptional state of intragraft T cells, BALB/c donor hearts were transplanted into CSB-treated B6 WT or *Irf8+32^–/–^* recipients. Extravascular immune cells were isolated from allografts 14 days after transplant by flow cytometry, and scRNA-Seq was performed, yielding 12,580 high-quality cells (Supplemental Figure 3, A and B). Allografts transplanted into *Irf8+32^–/–^* recipients exhibited shifts in CD4^+^ and CD8^+^ T cell phenotype (Figure 1, I and J, and Supplemental Figure 3, C–E). We observed an increase in Rora^+^CD4^+^ effector T cells in allografts from *Irf8+32^–/–^* recipients. Rora, a key regulator of Th17 cells, has been implicated in colitis, in which it drives T cell infiltration, activation, and prevention of apoptosis (5). Moreover, we observed marked reduction in a CD8^+^ T cell subset expressing *Tcf7*, *Xcl1*, and immunoregulatory genes (*Cd200*, *Cd160*, *Lag3*) in allografts from *Irf8+32^–/–^* recipients. *Xcl1* is secreted by CD8^+^ T cells and is a ligand for *Xcr1*, a cDC1-specific receptor that regulates antigen presentation, regulatory T cell activation, and prevents intestinal inflammation (6). Pathway analysis revealed upregulation of IL-1, IL-5, TNF, and CD40L signaling in T cells from allografts transplanted into *Irf8+32^–/–^* recipients and enhanced immunoregulatory responses and T cell apoptosis in WT recipients (Figure 1K).

Collectively, we demonstrate that cDC1s expand in response to CSB and are essential for long-term allograft acceptance. CSB facilitates recruitment of immunoregulatory cDC1s, which modulate T cell phenotypes. CSB represents a tractable approach to achieve organ transplant tolerance in the clinical setting. Unlike other tolerance protocols, CSB does not necessitate exposure of the recipient to donor cells or tissues and instead only involves perioperative treatment with CTLA4-Ig and anti-CD40L antibodies. Identification of cDC1s as a key cell involved in cardiac allograft acceptance provides a critical clue regarding underlying mechanisms. Future studies dissecting tolerogenic cDC1 effector mechanisms may lead to improved CSB regimens, methodologies to measure CSB efficacy, and platforms to predict posttransplant outcomes.

For detailed methods, information regarding sex as a biological variable, statistics, study approval, data availability, author contributions, and acknowledgments, see the Supplemental Methods.

## Supplementary Material

Supplemental data

Supporting data values

## Figures and Tables

**Figure 1:**
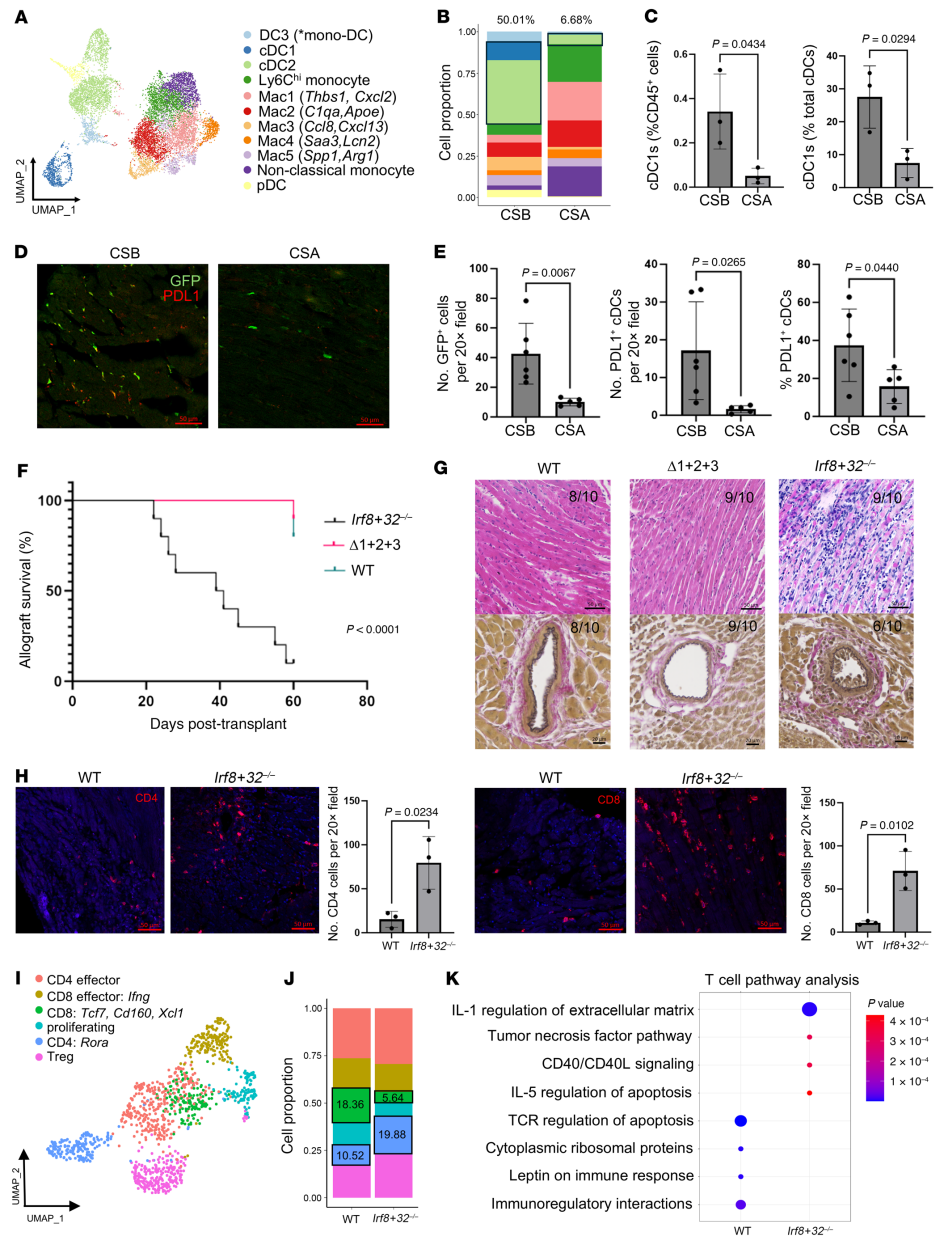
Type 1 classical DCs are necessary for cardiac allograft acceptance and modulate T cell phenotypes. (**A**) scRNA-Seq UMAP and (**B**) composition plot of mononuclear phagocytes sorted from allografts 7 days after transplantation of BALB/c hearts into CSB- (*n* = 3) or CSA-treated (*n* = 3) B6 WT mice. (**C**) Flow cytometry quantification of cDC1s within allografts of CSB- (*n* = 3) and CSA-treated (*n* = 3) B6 WT recipients at 7 days after transplant. (**D**) Immunostaining of graft-infiltrating cDCs (GFP^+^) and PDL1 in CSB- (*n =* 6) and CSA-treated (*n* = 5) B6 *Zbtb46^gfp/+^* recipients of BALB/c hearts at 7 days after transplant. Scale bar: 50 μm. (**E**) Quantification of GFP^+^ and PDL1^+^ cells in CSB- (*n* = 6) and CSA-treated (*n* = 5) allografts at 7 days after transplant into B6 *Zbtb46^gfp/+^* mice. (**F**) Kaplan-Meier survival curves of BALB/c hearts after transplantation into CSB-treated B6 WT, Δ1+2+3, and *Irf8+32^–/–^* mice (*n* = 10 per condition). (**G**) Histology (H&E, Verhoeff-Van Gieson elastin stain) of allografts from B6 WT, Δ1+2+3, and *Irf8+32^–/–^* recipients at 60 days after transplant (WT, Δ1+2+3) or time of rejection (*Irf8+32^–/–^*). Fractions in the top right corner indicate the number of samples with histology matching the representative image out of the total samples in each cohort. Scale bar: 50 μm (top); 20 μm (bottom). (**H**) Immunostaining of CD4^+^ and CD8^+^ T cells in allografts of B6 *Irf8+32^–/–^* (*n =* 3) and WT (*n* = 3) recipients at 14 days after transplant. Scale bar: 50 μm. (**I**) scRNA-Seq UMAP and (**J**) composition plot of subclustered T cells B6 WT (*n* = 3) and *Irf8+32^–/–^* (*n* = 3) recipients. (**K**) Pathway analysis of genes differentially expressed in T cells.

## Version 1 07/08/2025

In-Press Preview

## Version 2 09/16/2025

Electronic publication
