# Supplementary material for: Type 1 classical dendritic cells govern long-term cardiac allograft acceptance
Source: J Clin Invest. 2025 Jul 8;135(18):e192811. doi: 10.1172/JCI192811 (PMC12435831; doi:10.1172/JCI192811)
Supplement: Supplemental data [file jci-135-192811-s196.pdf]

Sex as a biological variable: The present study utilized equal numbers of male and female mice in each experimental group, and we report no differences in findings between sexes.

Animals: Eight- to twelve-week-old age- and sex-matched mice were used for all transplantation experiments. C57BL/6J (B6) (strain # 000664), B6 Zbtb46$^{gfp/+}$ (strain # 027618) and BALB/cJ (strain # 000651) mice were obtained from the Jackson Laboratories (Bar Harbor, ME). B6 *Irf8* +32$^{-/-}$ and B6 Δ1+2+3 mice were provided by Dr. Kenneth Murphy and were generated as previously described (3, 4). Mice were housed in temperature-controlled rooms (20–22°C) and humidity (40–60%) with a 12-hour light/dark cycle and provided ad libitum access to standard rodent chow and water.

Heart transplantation and tolerance induction strategy: Heart grafts were harvested from donor mice and transplanted heterotopically into recipients following 60 minutes of cold (4°C) ischemia. In brief, mice were anesthetized intraperitoneally with a mixture of ketamine (80-100 mg/kg) and xylazine Hcl (8-12 mg/kg) and were maintained on 1%-2% isoflurane gas as required. As indicated, 10 mg/kg of Cyclosporine (CSA) (Sigma-Aldrich, CAS Number 59865-13-3) was administered subcutaneously every day for 7 days following transplant. For mice receiving costimulation blockade, 250 μg anti-CD40L antibody was administered intraperitoneally at the time of transplant and 200 μg CTLA4-Ig intraperitoneally was administered on day 2 post-transplant. In an additional model utilized for scRNA-seq reference mapping, recipient mice received 200 ug CTLA4-Ig intraperitoneally on days 0, 2, 4 and 6 post-transplant. After transplantation, grafts were palpated daily. Cessation of a palpable heartbeat, confirmed by visual inspection, indicated rejection of a cardiac allograft.

Flow cytometry and sorting for single cell RNA sequencing: Single cell suspensions were generated from phosphate buffered saline (PBS)-perfused hearts by finely mincing and digesting hearts in Dulbecco's Modified Eagle Medium (DMEM) with Collagenase IV (Sigma C5138) 4500 U/mL in DMEM, Hyaluronidase 1 (Sigma H3506) 2400 U/mL in DMEM, and

DNAse I (Sigma D4527-40KU) 6000 U/mL in DMEM for 45 minutes at 37°C. To deactivate the digestion enzymes, samples were washed with Hank's Balanced Salt Solution (HBSS) that was supplemented with 2% Fetal Bovine Serum (FBS) and 0.2% Bovine Serum Albumin (BSA) and filtered through 40 μm cell strainers. Red blood cell lysis was performed with ACK lysis buffer (Thermo Fisher Scientific) for five minutes at room temperature. Samples were washed with DMEM and resuspended in 100 μL of FACS buffer (PBS with 2% FBS and 2 mM EDTA). Cells were stained with monoclonal antibodies at 4°C for 30 minutes in the dark. A complete list of antibodies is provided in the table below. Samples were washed in FACS buffer and a final resuspension was made in 300 μL FACS buffer.

For single cell RNA sequencing (scRNA-seq) sorting using *Zbtb46*$^{gfp/+}$ heart transplant recipients at 7 days post-transplant, flow cytometric sorting was performed on a BD FACS Melody platform. Cells were first gated as CD45^{+} and then sorted by cDCs (GFP^{+}), or macrophages and monocytes (CD45^{+}Ly6G^{-}CD64$^{+/-}$Ly6C$^{hi/low}$). Cells from n=3 CSA-treated *Zbtb46*$^{gfp/+}$ recipients and n=3 CSB-treated *Zbtb46*$^{gfp/+}$ recipients were sorted into 300 μL cell resuspension buffer (0.04% BSA in PBS). Collected cells were centrifuged as above, pooled across cohorts (CSA versus CSB), and resuspended in collection buffer to a target concentration of 1,000 cells/μL. Cells were counted on a hemocytometer before proceeding with the 10x Genomics protocol. cDNA construction and library preparation were performed for 4 libraries (CSA-treated GFP^{+}, CSA-treated macrophage/monocytes, CSB-treated GFP^{+}, and CSB-treated macrophages/monocytes). The following table includes the antibodies used for this sequencing experiment.

Antibody	Source	Identifier
CD45- PerCP-Cy5.5, clone 30-F11	BioLegend	Cat 103132
CD11b- BV421, clone M1/70	BioLegend	Cat 101251
Ly6G- PE, clone 1A8	BioLegend	Cat 127607
CD3- PE, clone 17A2	BioLegend	Cat 100205
CD19- PE, clone 6D5	BioLegend	Cat 115508
CD64- APC clone X54-5/7.1	BioLegend	Cat 139306
Ly6C- BV510 clone HK1.4	BioLegend	Cat 128033

For scRNA-seq of allografts 14 days after transplantation into *Irf8* $+32^{-/-}$ and WT recipients, flow cytometric sorting was performed on a BD FACS Melody platform. 5 minutes prior to harvest, a CD45 monoclonal antibody was administered intravenously (IV) to mice for flow cytometric exclusion of any immune cells residing within the graft vasculature. Cells were sorted as $CD45^{+}$ $DAPI^{-}$ $Ly6G^{-}$ CD45 IV^{-}. Cells from n=3 *Irf8* $+32^{-/-}$ and n=3 WT recipients were sorted into 300 μL cell suspension buffer (0.04% BSA in PBS). Collected cells were centrifuged as above, pooled across cohorts (*Irf8* $+32^{-/-}$ versus WT), and resuspended in collection buffer to a target concentration of 1,000 cells/μL. Cells were counted on a hemocytometer before proceeding with the 10x Genomics protocol. cDNA construction and library preparation were performed for 2 libraries (*Irf8* $+32^{-/-}$ and WT) as described in the following section. The following table includes the antibodies used for this sequencing experiment.

Target	Source	Identifier
CD45- PerCP-Cy5.5, clone 30-F11	BioLegend	Cat 103132
CD45 (administered IV)- APC, clone 30-F11	BioLegend	Cat 103112

Ly6G- PE, clone 1A8	BioLegend	Cat 127607
Live/Dead DAPI	BioLegend	Cat 422801

Single cell RNA sequencing preparation and analysis: Single cell captures and library preparation were performed using chemistry version 3 (V3) according to 10x Genomics instructions. Cells were loaded into 10x Genomics microfluidics chip G for encapsulation with their proprietary barcoded gel beads in the 10x Genomics Chromium X controller. Single-cell libraries were constructed according to manufacturer's instructions with index labeling using the Dual Index Kit TT set A. This allows for library pooling into one lane for sequencing on the Illumina NovaSeq6000 platform with a targeted depth of 75,000 reads per cell.

Single cell RNA sequencing data alignment, quality control and cell type annotation: Raw FASTQ files were aligned to the GRCm38 reference genome (v) using Cell Ranger (10x Genomics, v6.1). Subsequent quality control, normalization, dimensional reduction, and clustering were performed with Seurat v4.0. Cells were filtered using the following thresholds: 500 < nFeature_RNA < 7000; nCount_RNA < 30,000; percent mitochondrial reads < 10. For the first scRNA-seq experiment (CSA versus CSB), 23547 cells were recovered prior to QC filtering. A total of 14524 cells that passed QC filtering were used for subsequent analysis. For the second scRNA-seq experiment (*Irf8* +32$^{-/-}$ versus WT), 15351 cells were recovered prior to QC filtering, and a total of 12580 cells that passed QC filtering were used for subsequent analysis. Raw RNA counts from cells that met these criteria were normalized and scaled using SCTransform regressing out percent mitochondrial reads and nCount_RNA. Principal component analysis (PCA) was performed on the normalized RNA counts, and the number of PCs used for downstream clustering was dependent on the following criteria: PCs cumulatively explain > 99% of the variation present in the data and percent variation associated with the PCs

is less than 1%. Weighted nearest neighbor clustering (WNN) was performed with the significant RNA PCs directly without PCA as previously outlined with the FindMultiModalNeighbors function in Seurat. Subsequently, a uniform manifold approximation (UMAP) embedding was constructed and FindClusters was used to cluster cells using the SLM modularity optimization algorithm in an unbiased manner. Clustering was performed for a suite of different resolutions (0.1-0.8 at 0.1 intervals) and differential gene expression testing was performed using the FindAllMarkers function and a Wilcoxon Rank Sum test with a logFC cut-off of 0.25 and a min.pct cut-off of 0.1. Genes were deemed statistically significant if adjusted p-value < 0.05 and absolute(log2FC) > 0.5.

Clusters were annotated using canonical gene markers and subsequent dot plots (RNA) were created to assess clean separation of clusters into distinct cell types. Previously identified marker genes were also plotted on the UMAP object to further validate cluster annotations.

Pathway Analysis: Statistically significant differentially expressed (DE) genes were used to perform pathway analysis via EnrichR (https://maayanlab.cloud/Enrichr/). Pathway enrichment values were downloaded as .txt files and plots generated with EnrichPlot and EnrichR packages in Seurat.

T-cell states analysis: To cluster T-cells into distinct cell states, we subsetted the T-cells from the global scRNA-seq object, re-normalized, computed PCAs, computed UMAPs, and clustered data at a range of resolutions. DE analysis was then used to identify marker genes for each cell state and a dot plot to assess clustering separation. Using the top marker genes we calculated gene set z-scores and plotted them in UMAP space.

Histology: Upon harvest, hearts were perfused with 1X PBS and then placed in 4% PFA overnight at 4°C. After 24 hours, hearts were rinsed with 1X PBS and placed in tissue histology cassettes in 70% ethyl alcohol for paraffin embedding. Paraffin blocks were sectioned on a microtome with a clean blade at 5 μm thickness and transferred onto charged microscope slides for staining. Slides were baked at 60°C for 1 hour and then passed through multiple exchanges of xylenes/ethanol for dehydration/rehydration prior to staining with Hematoxylin and Eosin (H&E). H&E staining was performed using Mayer's Hematoxylin-Lillie's Modification (Agilent Dako, Cat # S3309), Dako bluing buffer (Agilent Dako, Cat # CS702), and Eosin Y Solution Alcoholic (Sigma-Aldrich, Cat # HT110116). Verhoeff-Van Gieson (VVG) staining was performed using an Elastic, Verhoeff Stain Kit (Newcomer Supply, Part # 9116B) per manufacturer's instructions. After staining, tissues were dehydrated in ethyl alcohol, cleared in xylenes, and then mounted with Cytoseal (Epredia, Cat # 22-050-262) medium prior to placement of no. 1.5 coverslips. Images of H&E and VVG-stained tissues were obtained on a Zeiss Axioscan Z1 automated slide scanner and a Zeiss LSM 700 confocal microscope.

Immunofluorescence: Paraffin-embedded heart tissue samples were fixed for 24 hours at 4°C in 4% paraformaldehyde, washed in 1X PBS, and embedded in paraffin. Paraffin-embedded sections were cut at 5 μm thickness using a microtome. Slides were baked at 60°C for 1 hour and then passed through multiple exchanges of xylenes/ethanol for dehydration/rehydration. Slides underwent methanol treatment (10% MeOH + 3% H_2O_2) for 20 minutes at room temperature followed by 3X TBS-T washes (5 min each). Antigen retrieval was performed using the AR6 buffer (Akoya Biosciences, Cat # AR600250ML) for 15 minutes in the microwave and then cooled to room temperature. Tissue sections were marked with a hydrophobic pen and blocked in 10% BSA in TBS-T for 30 minutes at room temperature. Slides were then stained with the primary antibody diluted in 10% BSA in TBS-T overnight at 4 °C (CD8 1:500 dilution (Cell Signaling Technology Cat # 98941S), CD4 1:200 dilution (Abcam Cat # EPR6855), Foxp3

1:100 dilution (Invitrogen Cat # 13-5773-82)). Next, the primary antibody was detected using Opal Polymer HRP Ms + Rb (Akoya Biosciences Opal Multicolor IHC system). The PerkinElmer Opal Multicolor IHC system was utilized to visualize antibody staining per manufacturer's protocol.

GFP and PDL1 staining of allografts collected from Zbtb46$^{gfp/+}$ mice was performed on frozen tissue sections. At the time of tissue collection, hearts were perfused with 1X PBS and placed in 4% PFA overnight at 4°C. Hearts were rinsed with PBS x 3 and infiltrated with 30% sucrose (in PBS) overnight at 4°C. Hearts were embedded in Optimal Cutting Temperature (O.C.T.) Compound (Fisher HealthCare Tissue Plus O.C.T. Compound Cat # 4585) and frozen at -80°C. 10 µm sections were obtained using a Leica Cryostat. Sections were washed in TBS and stained in 10% FBS in TBS-T (0.05% Tween-20) blocking solution with primary antibody (anti-GFP 1:2000 dilution (Abcam Cat # ab13970), PDL1 1:200 dilution (Abcam Cat # ab205921)) overnight at 4°C in a humidified environment. After washing, the secondary antibody (Alexa Fluor 488 goat anti-chicken (Cat # A11039 1:200 dilution), Alexa Fluor 555 goat anti-rabbit (Cat # A21428 1:200 dilution)) was added to blocking buffer and sections were stained for 60 minutes at room temperature protected from light. DAPI mounting, anti-fade solution (Fluoroshield with DAPI, Sigma-Aldrich Cat # F6057) was added immediately prior to placement of no. 1.5 coverslips.

Following staining, slides were imaged using a Zeiss Axioscan Z1 automated slide scanner and a Zeiss LSM 700 confocal microscope. Image processing was performed using Zen Blue and Zen Black (Zeiss). 3 random sections of tissue were quantified for presence of fluorescent signal (CD4, CD8, GFP, PDL1, Foxp3) colocalized with DAPI. Each dot on quantification bar graphs represents the average cell number quantified from 3 random regions of tissue per allograft.

Statistics: Cell quantification of immunostained tissues was performed on random sections of tissues, blinded to condition and genotype. For analysis of immunofluorescence, two tailed t-test assuming equal variance was performed in Prism. For analyzing allograft survival probability, a Kaplan Meier survival curve was generated in Prism and analyzed for significance with a Log-rank (Mantel-Cox) test. P values of < 0.05 were deemed statistically significant.

Study approval: All experiments conformed to the Guide for the Care and Use of Laboratory Animals from the National Institute of Health (NIH publication no. 85–23, revised 1996). Animal study protocols were approved by Animal Care and Use Committee at Washington University in St. Louis.

Data availability: Raw and processed scRNA-seq data are available from the Gene Expression Omnibus repository (accession number GSE296427). Values for all data points in graphs are reported in the Supporting Data Values file. The other data that support the findings of this study are available from the corresponding author on reasonable request.

Code availability: The R scripts used to analyze the scRNA-seq datasets in this study are available at https://github.com/owenmacee/mowen-lavine2025/tree/main.

Acknowledgments: KJL is supported by the Washington University in St. Louis Rheumatic Diseases Research Resource-Based Center grant (NIH P30AR073752), the National Institutes of Health [R01 HL138466, R01 HL139714, R01 HL151078, R01 HL161185, R35 HL161185], Leducq Foundation Network (#20CVD02), Burroughs Welcome Fund (1014782), and Children's Discovery Institute of Washington University and St. Louis Children's Hospital (CH-II-2015-462, CH-II-2017-628, PM-LI-2019-829), Foundation of Barnes-Jewish Hospital (8038-88), and generous gifts from Washington University School of Medicine. DK is supported by NIH (P01AI116501 and R01 HL094601), Veterans Administration Merit Review (1I01BX002730), the Cystic Fibrosis Foundation and the Foundation for Barnes-Jewish Hospital. MCO was

supported by the NIH T32 AI007163. We thank the Genome Technology Access Center at the McDonnell Genome Institute at Washington University School of Medicine for help with genomic analysis. The Center is partially supported by NCI Cancer Center Support Grant #P30 CA91842 to the Siteman Cancer Center. We also thank Dr. Kenneth Murphy for his generous contribution of the *Irf8* $+32^{-/-}$ and Δ1+2+3 mice. We would also like to acknowledge the support of the Washington University Center for Cellular Imaging (WUCCI), which is supported in part by Washington University School of Medicine, The Children's Discovery Institute of Washington University and St. Louis Children's Hospital (CDI-CORE-2015-505 and CDI-CORE-2019-813) and the Foundation for Barnes-Jewish Hospital (3770) for access to imaging resources and valuable technical assistance. We would also like to thank the Center for Cardiovascular Research (CCR) microscopy center for the use of the Zeiss LSM 700 confocal microscope.

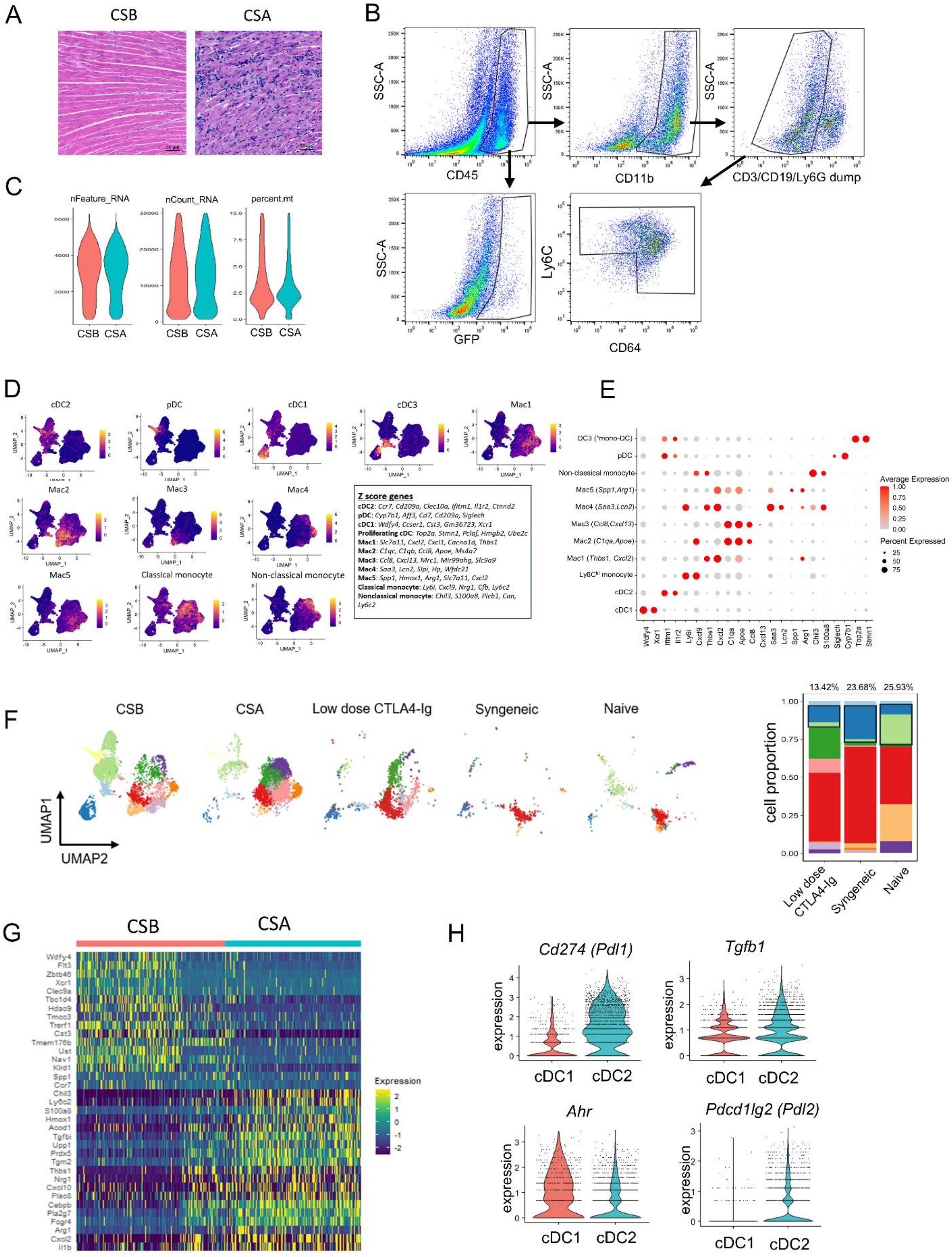
A
CSB
CSA
B
SSC-A
CD45
CD11b
CD3/CD19/Ly6G dump
GFP
Ly6C
CD64
C
nFeature_RNA
nCount_RNA
percent.mt
CSB CSA
D
cDC2
pDC
cDC1
cDC3
Mac1
Mac2
Mac3
Mac4
Mac5
Classical monocyte
Non-classical monocyte
UMAP_1
UMAP_2
Z score genes
cDC2: Ccr7, Cd209a, Clec10a, Ifitm1, Il1r2, Ctnnd2
pDC: Cyp7b1, Aff3, Cd7, Cd209a, Siglech
cDC1: Wdfy4, Ccser1, Cst3, Gm36723, Xcr1
Proliferating cDC: Top2a, Stmn1, Pclaf, Hmgb2, Ube2c
Mac1: Slc7a11, Cxcl2, Cxcl1, Cacna1d, Thbs1
Mac2: C1qc, C1qb, Ccl8, Apoe, Ms4a7
Mac3: Ccl8, Cxcl13, Mrc1, Mir99ahg, Slc9a9
Mac4: Saa3, Lcn2, Slpi, Hp, Wfdc21
Mac5: Spp1, Hmox1, Arg1, Slc7a11, Cxcl2
Classical monocyte: Ly6i, Cxcl9, Nrg1, Cfb, Ly6c2
Nonclassical monocyte: Chil3, S100a8, Plcb1, Can, Ly6c2
E
DC3 (*mono-DC)
pDC
Non-classical monocyte
Mac5 (Spp1,Arg1)
Mac4 (Saa3,Lcn2)
Mac3 (Ccl8,Cxcl13)
Mac2 (C1qa,Apoe)
Mac1 (Thbs1, Cxcl2)
Ly6C^hi monocyte
cDC2
cDC1
Average Expression
Percent Expressed
Wdfy4 Xcr1 Ifitm1 Il1r2 Ly6i Cxcl9 Thbs1 Cxcl2 C1qa Apoe Ccl8 Cxcl13 Saa3 Lcn2 Spp1 Arg1 Chil3 S100a8 Siglech Cyp7b1 Top2a Stmn1
F
CSB
CSA
Low dose CTLA4-Ig
Syngeneic
Naive
UMAP1
UMAP2
13.42% 23.68% 25.93%
cell proportion
Low dose CTLA4-Ig
Syngeneic
Naive
G
CSB
CSA
Wdfy4 Flt3 Zbtb46 Xcr1 Clec9a Tbc1d4 Hdac9 Tmcc3 Trerf1 Cst3 Tmem176b Ust Nav1 Klrd1 Spp1 Ccr7 Chil3 Ly6c2 S100a8 Hmox1 Acod1 Tgfbi Upp1 Prdx5 Tgm2 Thbs1 Nrg1 Cxcl10 Plac8 Cebpb Pla2g7 Fcgr4 Arg1 Cxcl2 Il1b
Expression
H
Cd274 (Pdl1)
Tgfb1
Ahr
Pdcd1lg2 (Pdl2)
expression
cDC1 cDC2

Supplemental Figure 1: A) Histology of BALB/c hearts 7 days post-transplant into B6 WT mice treated with CSB (n=6) or CSA (n=5). **B)** Flow cytometry gating scheme for scRNA-seq of APCs ($CD45^{+}GFP^{+}$; $CD45^{+}CD11b^{+}CD3^{-}CD19^{-}Ly6G^{-}CD64^{+/-}Ly6C^{hi/lo}$) sorted from allografts of *Zbtb46*^{gfp} recipient mice at 7 days post-transplant, treated with CSB (n=3) or CSA (n=3) treatment. **C)** Quality control metrics including number of RNA features, RNA counts, and percent mitochondrial reads (500 < nFeature_RNA < 7000; nCount_RNA <30,000; percent mitochondrial reads <10) of 14524 high-quality cells post-filtering for downstream scRNA-seq analysis. **D)** Z-scores generated from the top 5 statistically significant (avg. logFC >0.5, adjusted P value <0.05, Bonferroni correction) differentially expressed genes per cell state plotted into the UMAP projection. **E)** Dot plot of the top 2 statistically significant (avg. logFC >0.5, adjusted P value <0.05, Bonferroni correction) differentially expressed genes in cell states. **F)** UMAP projections of reference-mapped control samples (Low dose CTLA-4Ig allografts, syngeneic allografts, naïve hearts) and composition plot of relative cell proportions. Also depicted is the percentage of classical dendritic cell proportion in each condition. **G)** Heat map of top statistically significant (avg. logFC >0.5, adjusted P value <0.05, Bonferroni correction) differentially expressed genes in allografts after transplantation into CSB- relative to CSA-treated recipients. **H)** Violin plots of immunoregulatory gene expression in cDC1s and cDC2s within CSB-treated allografts.

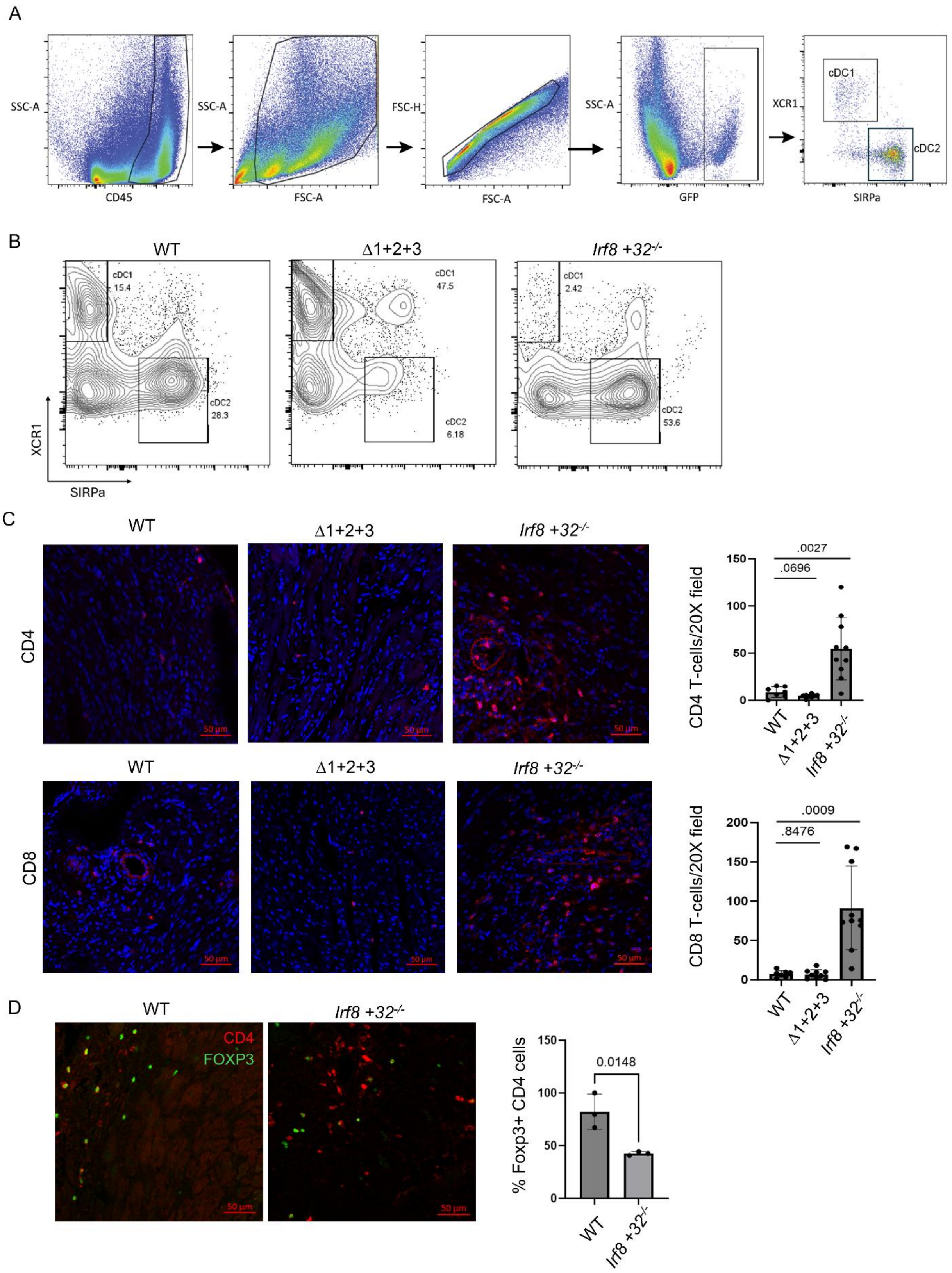
A
SSC-A
CD45
SSC-A
FSC-A
FSC-H
FSC-A
SSC-A
GFP
XCR1
SIRPa
cDC1
cDC2
B
WT
Δ1+2+3
Irf8 +32-/-
cDC1
15.4
cDC2
28.3
cDC1
47.5
cDC2
6.18
cDC1
2.42
cDC2
53.6
XCR1
SIRPa
C
WT
Δ1+2+3
Irf8 +32-/-
CD4
CD8
50 µm
CD4 T-cells/20X field
.0027
.0696
CD8 T-cells/20X field
.0009
.8476
D
WT
Irf8 +32-/-
CD4
FOXP3
% Foxp3+ CD4 cells
0.0148

Supplemental Figure 2: A) Flow cytometry gating scheme to identify cDC1s ($Xcr1^+$ $Sirp\alpha^-$) and cDC2s ($Xcr1^-$ $Sirp\alpha^+$) from BALB/c hearts after transplantation into B6 *Zbtb46*$^{gfp/+}$ recipients. **B)** Flow cytometry plot depicting cDC1 and cDC2 proportions in splenocytes isolated from B6 WT (n=10), Δ1+2+3 (n=10), and *Irf8* $+32^{-/-}$ (n=10) heart allograft recipients at 60 days post-transplant (WT, Δ1+2+3) or time of rejection (*Irf8* $+32^{-/-}$). **C)** Immunostaining and quantification of $CD4^+$ and $CD8^+$ T-cells in BALB/c allografts following transplantation into CSB-treated B6 WT, Δ1+2+3, and $Irf8^{+32-}/^-$ recipients (analyzed at 60 days post-transplant for WT and Δ1+2+3, or at time of rejection for $Irf8^{+32-}/^-$). Statistical analysis performed using a two-tailed unpaired t-test. Each dot represents the average number of cells quantified from 3 random regions per allograft. **D)** Immunostaining and quantification of $Foxp3^+$ $CD4^+$ T-cells in BALB/c allografts 14 days after transplantation into CSB-treated B6 WT (n=3) and *Irf8* $+32^{-/-}$ recipients (n=3). Each dot represents the average number of cells quantified from 3 random regions per allograft. Statistical analysis performed using a two-tailed unpaired t-test.

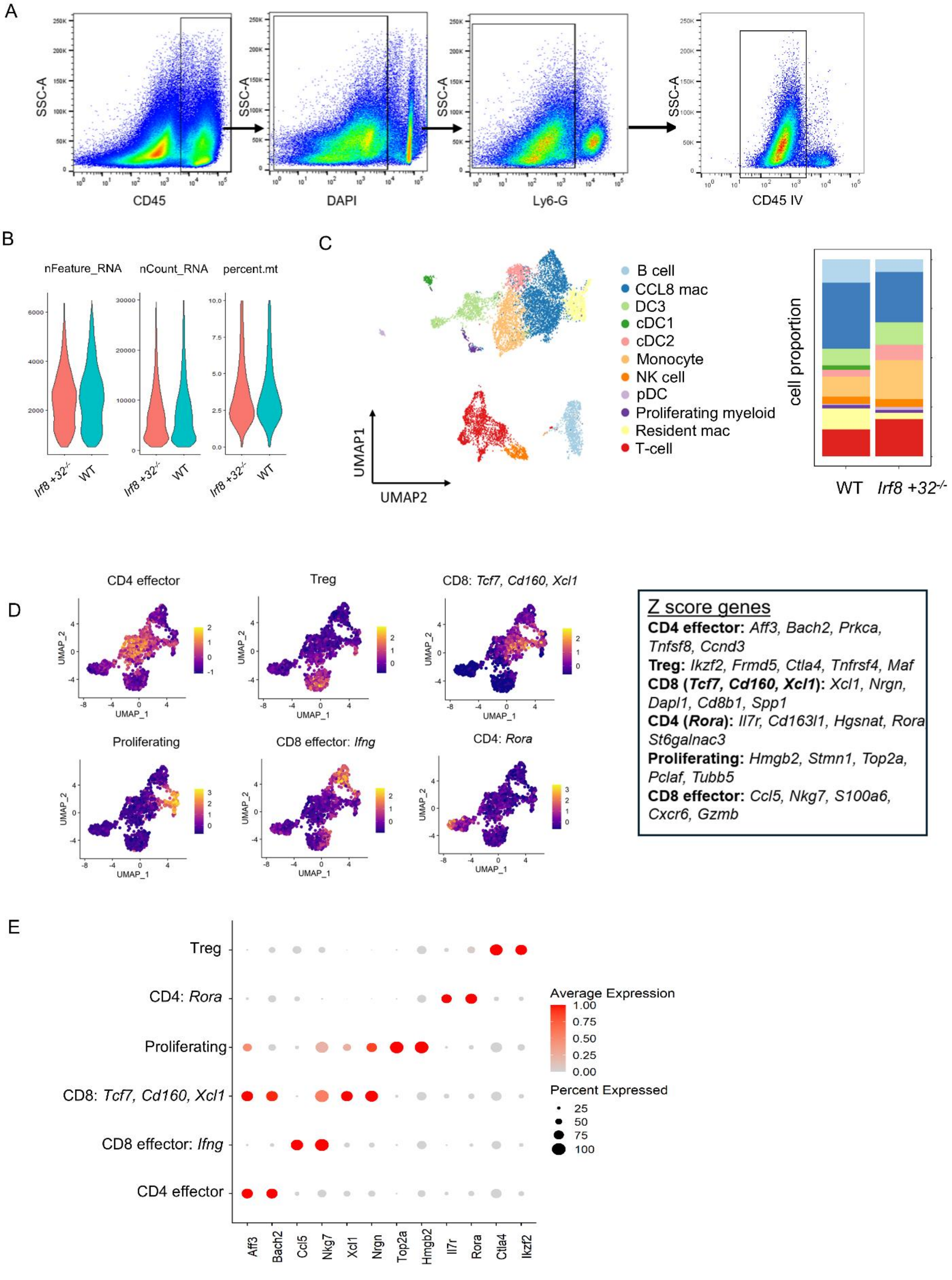
A
SSC-A
CD45
DAPI
Ly6-G
CD45 IV
B
nFeature_RNA
nCount_RNA
percent.mt
Irf8 +32-/-
WT
C
B cell
CCL8 mac
DC3
cDC1
cDC2
Monocyte
NK cell
pDC
Proliferating myeloid
Resident mac
T-cell
UMAP1
UMAP2
cell proportion
WT
Irf8 +32-/-
D
CD4 effector
Treg
CD8: Tcf7, Cd160, Xcl1
Proliferating
CD8 effector: Ifng
CD4: Rora
UMAP_1
UMAP_2
Z score genes
CD4 effector: Aff3, Bach2, Prkca, Tnfsf8, Ccnd3
Treg: Ikzf2, Frmd5, Ctla4, Tnfrsf4, Maf
CD8 (Tcf7, Cd160, Xcl1): Xcl1, Nrgn, Dapl1, Cd8b1, Spp1
CD4 (Rora): Il7r, Cd163l1, Hgsnat, Rora St6galnac3
Proliferating: Hmgb2, Stmn1, Top2a, Pclaf, Tubb5
CD8 effector: Ccl5, Nkg7, S100a6, Cxcr6, Gzmb
E
Treg
CD4: Rora
Proliferating
CD8: Tcf7, Cd160, Xcl1
CD8 effector: Ifng
CD4 effector
Average Expression
1.00
0.75
0.50
0.25
0.00
Percent Expressed
25
50
75
100
Aff3
Bach2
Ccl5
Nkg7
Xcl1
Nrgn
Top2a
Hmgb2
Il7r
Rora
Ctla4
Ikzf2

Supplemental Figure 3: A) Flow cytometry gating scheme for scRNA-seq of extravascular $CD45^+$ immune cells ($CD45^+DAPI^-Ly6G^-CD45IV^-$) sorted from BALB/c hearts 14 days after transplantation into CSB-treated B6 *Irf8* $+32^{-/-}$ (n=3) and WT (n=3) recipients. **B)** Quality control metrics including number of RNA features, RNA counts, and percent mitochondrial reads (500 < nFeature_RNA < 7000; nCount_RNA <30,000; percent mitochondrial reads <10) of 12580 high-quality cells post-filtering for downstream scRNA-seq analysis. **C)** UMAP and composition plot of extravascular immune cells in allografts 14 days after transplantation into B6 WT and *Irf8* $+32^{-/-}$ mice. **D)** Z-scores generated from the top 5 statistically significant (avg. logFC >0.5, adjusted P value <0.05, Bonferroni correction) differentially expressed genes per cell state plotted into the UMAP projection. **E)** Dot plot of the top 2 statistically significant (avg. logFC >0.5, adjusted P value <0.05, Bonferroni correction) differentially expressed genes in T-cell states.
